# Research on the Application of New Building Recycled Insulation Materials for Walls

**DOI:** 10.3390/polym16152122

**Published:** 2024-07-25

**Authors:** Yan Liu, Qinglong Zhao, Xiaohua Gu, Anyu Fan, Shangwen Zhu, Qingyong Su, Li Kang, Lizhi Feng

**Affiliations:** 1 School of Energy and Building Environment, Guilin University of Aerospace Technology, Guilin 541004, China; yanliu2019009@163.com (Y.L.); glzsw2337899@163.com (S.Z.); sqy@guat.edu.cn (Q.S.); 18078343109@163.com (L.K.); fenglz319@163.com (L.F.); 2 College of Civil Engineering and Architecture, Northeast Petroleum University, Daqing 163318, China; qinglong4497@163.com; 3 School of Material Science and Engineering, Qiqihar University, Qiqihar 161006, China; fan18783442810@163.com

**Keywords:** recycled polyurethane, buildings, insulation material, insulation wall, green and environmental protection

## Abstract

In this paper, a new type of recycled polyurethane material is used as a new type of wall insulation material, and the new building insulation wall made of this paper has high efficiency thermal insulation and energy-saving characteristics and also has certain environmental significance. The thermal conductivity of the new building cold insulation recycled polyurethane material is 0.023 W/(m·K), and the thermal conductivity of the new building insulation wall prepared is 0.297 W/(m·K). Compared with traditional double-sided plastered porous wall tiles, it can save 85.4% of energy consumption per square meter, with higher thermal insulation characteristics and economic benefits. The preparation of a new type of building insulation wall proposed in this paper provides a new and green way for wall insulation.

## 1. Introduction

### 1.1. Traditional Ordinary Building Wall

The traditional, ordinary building wall of the building is an important part of the building insulation; traditional insulation material is styrene as the insulation material PU (Polyurethane, referred to as PU) is a polymer composed of organic units connected by urethane, the full name is polyurethane. This polymer compound is composed of polyisocyanates and polyhydroxy polymers, which have excellent mechanical properties and plasticity [1,2,3]. PU is a high-performance thermal insulation material; has attracted worldwide attention and has rapidly developed since it was first prepared by Otto Bayer in Germany in 1937 [4]. The application of PU in the construction field is particularly prominent, and its rigid foam PU material has excellent thermal insulation performance and is one of the main materials for building insulation layers. In addition, PU material has high strength and toughness, can withstand large external forces, is not easy to break, and has excellent mechanical properties. Products with different hardness and elasticity can be prepared by changing the formula and process conditions to meet various needs and have strong plasticity. PU has little impact on the environment during production and use and meets environmental protection requirements [5,6,7,8]. However, the gradual expansion of the application field of a large number of PU has led to a serious aggravation of the pollution of waste materials, which are difficult to degrade, which not only brings the problem of solving the pollution of waste materials to enterprises but also causes significant pollution [9]. Therefore, a quick way to protect the environment is to reuse waste.

In this paper, recycled polyurethane (RPU) is used as a new type of material to replace the traditional building wall insulation material, and the performance of the new material is evaluated by comparing the impact of the new material with the traditional wall insulation material on building energy consumption.

### 1.2. Traditional Wall Materials

Ordinary building walls are an important part of buildings, which not only bear the function of supporting the weight of the structure but also have a variety of properties such as sound insulation, heat insulation and fire protection [10]. According to the different wall materials, building walls can be divided into brick walls, aerated concrete block walls, stone walls, and panel walls [11]. In architectural applications, different types of walls are widely used in residential, public, and commercial buildings according to their characteristics [12]. Concrete walls are often used as partition walls and load-bearing walls in ordinary residences to improve the stability of buildings. In commercial buildings, concrete walls are often used for exterior wall decoration and interior wall treatment to enhance the aesthetics and comfort of the building [13]. Building walls are an integral part of the building.Not only support the structure of the entire building but also meet the multiple functional needs of the building through various materials and construction methods. 

**Figure 1:**
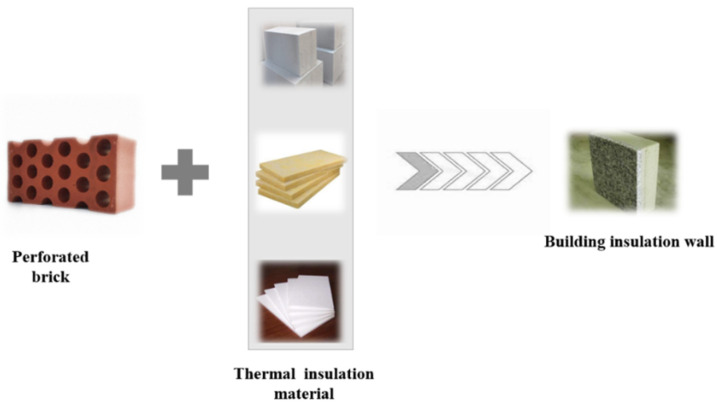
Flow chart of the production of building insulation wall.

Building wall insulation materials play multiple roles in the construction of buildings, such as improving the stability of building structures, heat insulation and insulation, sound insulation and noise reduction, beautifying the appearance of buildings, and preventing fire and moisture, and they are of great significance to meet people’s needs for building safety, comfort, and energy conservation [14,15,16]. The selection of building wall insulation materials needs to balance performance, cost, and construction difficulty. Traditional materials such as glass wool and rock wool have low thermal conductivity and good fire.Brick walls and aerated concrete are durable, stable, insulated, easy to construct and reduce noise [17,18,19]. In general, different building wall insulation materials have their own advantages and application scenarios, and when choosing, it is necessary to comprehensively consider the needs, costs, construction difficulties and other factors of the building. The combination of thermal insulation material and building wall forms the building thermal insulation wall with load-bearing capacity and good thermal insulation effect at the same time. The production flow chart of building thermal insulation walls is shown in Figure 1.

### 1.3. Energy Saving and Environmental Protection of PU Recycled Insulation Wall

**Figure 2:**
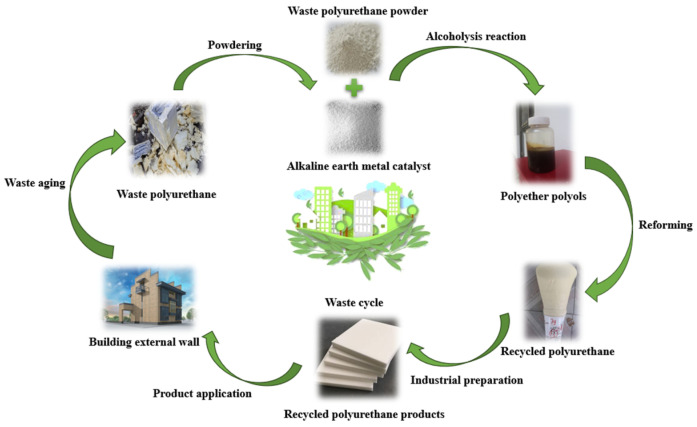
Schematic diagram of PU recycled insulation wall made from waste PU recycling.

In the early days, polyurethane waste was generally disposed of by burning and burying it. However, the waste smoke produced by the burning of waste materials seriously pollutes the atmosphere. Either way, it brings serious pollution to the environment and a waste of resources. In recent years, China’s energy consumption has been rising, based on the concept of “community with a shared future for mankind”. China has taken the lead in proposing a series of measures to optimize its energy structure. In 2020, General Secretary Xi Jinping proposed the “dual carbon” goal, and once the plan was proposed, it received responses from various industries. For the construction industry, the widespread implementation of green and energy-saving buildings is an important measure to achieve the “double carbon” goal. In this context, we recover waste PU through solid waste recycling and reuse waste PU through alcohololysis technology and regenerative foaming. For the construction industry, building wall insulation has always been a matter worthy of our attention. Ordinary wall insulation materials have limited energy reduction for buildings and have no environmental significance, and even the recycling of some waste insulation materials is still a big problem. Based on the current situation of the industry, we creatively use the recycled PU foam obtained from solid waste recycling as wall insulation materials and make a new type of building insulation wall with environmental protection significance. The schematic diagram of the PU recycled insulation wall prepared by recycling waste PU is shown in Figure 2 below.

### 1.4. Evaluation Indicators of Energy Consumption of Recycled Building Materials

When evaluating the energy consumption of a building, it is common to look at the overall energy consumption of the building, including the total energy consumption for the whole year, the energy consumption of refrigeration, the energy consumption of heating, the energy consumption of equipment, etc., and the gap in energy consumption that can be intuitively reflected is the gap in economic benefits [20]. This paper will integrate these two aspects to evaluate the building energy consumption of recycled building materials.

#### 1.4.1. Recycled Building Materials, Building Energy Consumption and Energy Saving per Unit Area

Recycled building materials: Building energy consumption and energy savings per unit area are calculated according to traditional calculation methods. In general, the evaluation of building energy consumption per unit area is an important method to evaluate the energy efficiency and energy-saving performance of buildings. It calculates and evaluates the ratio of a building’s energy consumption to its floor area, resulting in a building’s energy consumption level per unit area [21]. The building energy sub-standard evaluates different areas of energy consumption in the building, such as heating energy consumption, air conditioning energy consumption, lighting energy consumption, etc., which helps to understand in more detail the energy use of different aspects of the building. Building energy efficiency is mainly judged by comparing the annual electricity consumption of buildings due to the “carbon peak and carbon neutrality” policy stipulated by the state. Therefore, the environmental protection characteristics of the building wall are also attached to the evaluation of energy saving, as long as it is achieved by judging the carbon emission of the building wall. The energy consumption, energy savings, and environmental protection of the comprehensive building wall are compared, and the building insulation materials with the best comprehensive performance are compared.

#### 1.4.2. Software Simulation of Recycled Building Materials

In this paper, Tianzheng HVAC software (t20v7.0) is used to simulate the energy consumption of buildings made of recycled building materials, and the design code for heating, ventilation and air conditioning of civil buildings is used to comprehensively obtain the energy consumption of buildings through the human body, fresh air, lighting and equipment. By comparing a variety of common building insulation materials with building insulation walls, the thermal insulation capacity was analyzed. In addition, energy consumption, economic benefits, environmental benefits, etc., were analyzed. The performance of building materials and insulation walls was comprehensively evaluated.

## 2. Simulation Analysis of Recycled Building Materials

### 2.1. Preparation of PU Recycled Building Materials and Manufacturing Process of Building Insulation Walls

#### 2.1.1. Preparation of PU Recycled Building Materials

**Figure 3:**
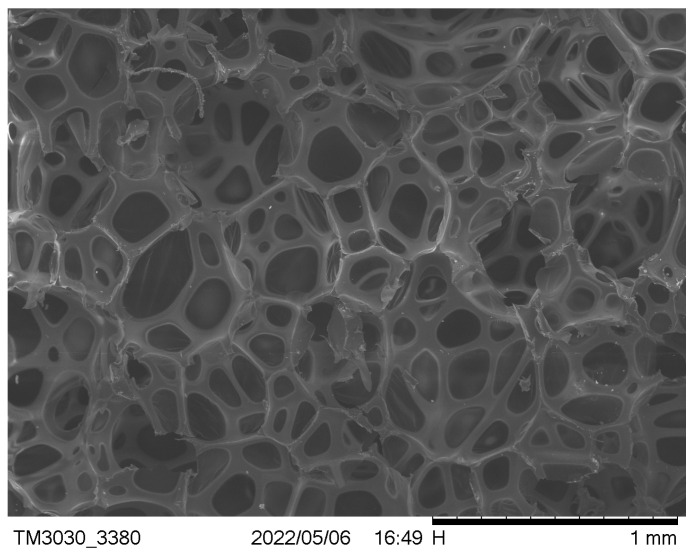
Polarizing microscope image of recycled foam prepared from waste PU.

The waste PU foam was successfully degraded by the two-component alcohololytic agents, propylene glycol and diethylene glycol, and the alkaline earth metal catalyst was added with 1.2 g, and the reaction condition was 180 °C for 2 h. 7 g of degraded recycled polyether polyol, 23 g of commercial polyether polyol 4110, 2.5 g of dimethyl silicone oil (DSO), 13 g of blowing agent (CAS number 287-92-3), 0.3 g of catalyst triethanolamine (TEA), and 0.1 g of dibutyltin dilaurate (DBTDL) in disposable plastic cups, followed by a cantilever mixer for 2 min of thorough mixing of the mixture. Immediately after mixing, 45 g of polyisocyanate (PAPI) is added to the homogeneous mixture and stirring is continued to prepare the foaming material. The prepared new chemical pipeline materials need to be left in a cool and dry place for 24 h to stabilize their performance, and then samples are made according to relevant test standards for subsequent testing. The compressive strength of PU foam obtained by foaming and regeneration of the degradation product is 0.413 MPa, the apparent density is 0.041 g/cm^3^, the thermal conductivity is 0.025 W/(m·K), and the adiabatic coefficient is 0.041 W/(m·K), which meets the national standard, and the sample bubble hole is relatively complete, the skeleton is relatively thick, and the insulation effect is better. The scanning electron microscope (SEM) diagram of RPU foam is shown in Figure 3; the bubble cells are uniform and complete, the skeleton is good, and it plays a good role in supporting the foam.

RPU materials have excellent corrosion and aging resistance and can perform stably in extreme environments, ensuring long-term performance without compromise. Its excellent electrical properties, high dielectric strength and high-temperature resistance provide a reliable safety barrier for electrical systems to prevent failures caused by insulation failure. With its unique closed-cell structure and high gas barrier ability, RPU rigid foam exhibits insulation performance that surpasses traditional materials such as polystyrene board and rock wool and maintains an efficient insulation effect for a long time. In addition, polyurethane insulation is insensitive to temperature and humidity changes, and its stable performance adapts to various climatic conditions, ensuring reliable insulation protection for buildings and equipment in extreme environments and ensuring safe operation.

#### 2.1.2. Manufacturing Process of Waste PU Recycled Building Insulation Wall

**Figure 4:**
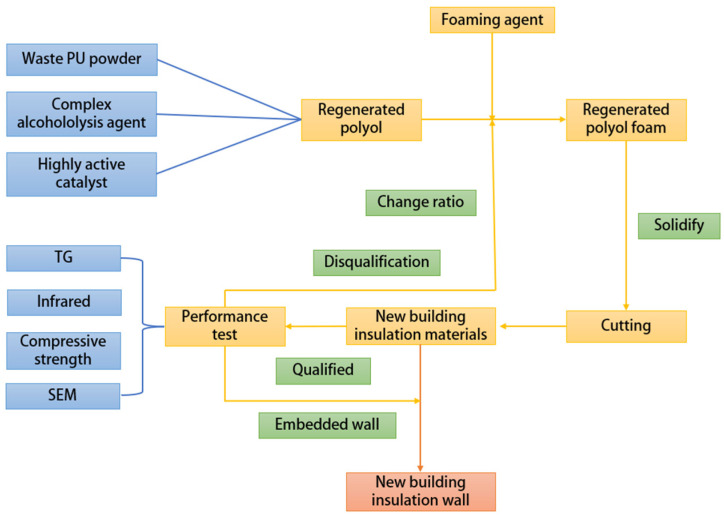
Flow chart of waste polyurethane regeneration of new building insulation materials.

The recycled PU foam with qualified performance is embedded into the double-sided plastered porous brick wall to make a new building insulation wall, with a total thickness of 35 cm, of which the thickness of the recycled PU insulation layer is 70 mm, and the total heat transfer coefficient of the recycled PU insulation wall is K = 0.297 W/(m·K). The process of polyurethane regeneration into a new type of building insulation wall is shown in Figure 4.

### 2.2. Project Overview

**Figure 5:**
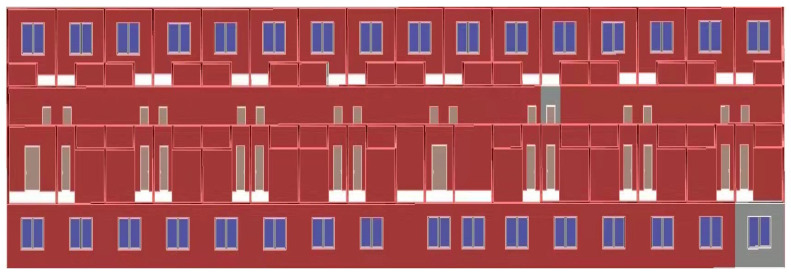
Schematic diagram of a certain layer of the building.

Using Tianzheng HVAC software to simulate a multi-story hotel, the building is located in Guilin City, Guangxi Province, with a total construction area of about 2025.05 m^2^; taking a certain floor of the building as the simulation object, the building floor height is 3.5 m, and the rooms are arranged as guest rooms, corridors, etc. The environmental parameters are calculated according to the environmental parameters of Guangxi Province, and the building type is defined as a residential area without considering the influence of surrounding building parameters. The schematic diagram of a certain layer of the building is shown in Figure 5.

### 2.3. Wall Structure and Parameter Setting

**Figure 6:**
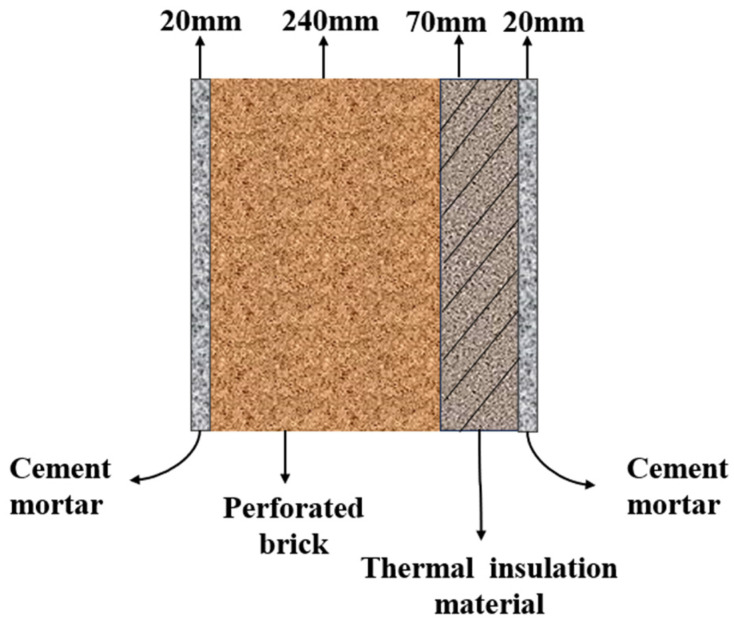
Structural diagram of building insulation wall.

The structure of the building insulation wall is shown in Figure 6; left to right are cement mortar, perforated brick walls, building insulation and cement mortar. The thickness of cement mortar is 20 mm, the thickness of porous brick wall is 240 mm, and the thickness of insulation material is 70 mm.

There are five options for the exterior wall, which are:

(1) Perforated brick wall, painted inside and outside, ordinary brick wall, δ = 280 mm.
(2) Perforated brick wall, painted inside and outside, and the insulation material is aerated concrete, δ = 350 mm.
(3) Perforated brick wall, stucco inside and outside, insulation material is polystyrene board, δ = 350 mm.
(4) Perforated brick wall, painted inside and outside, the insulation material is rock wool, δ = 350 mm.
(5) Perforated brick wall, painted inside and outside, and the insulation material is a new type of building insulation recycled PU material, δ = 350 mm.

Interior walls: Concrete perforated brick walls, δ = 200, K = 1.86 W/(m·K), β = 0.3;

Exterior windows: single-layer steel windows, K = 2.6 W/(m·K). The effective area coefficient of the window X_g_ = 0.85, the location correction coefficient X_d_ = 1, take the 6 mm thick ordinary glass, the shading coefficient C_S_ = 1, the light-colored curtain is selected, and the shading coefficient C_n_ = 1;

The indoor and outdoor parameters of the building are selected with reference to the architectural design code of Guangxi Province. The indoor and outdoor parameters are shown in Table 1.

## 3. Result Analysis

### 3.1. Comparison of Thermal Conductivity of Different Thermal Insulation Walls

**Figure 7:**
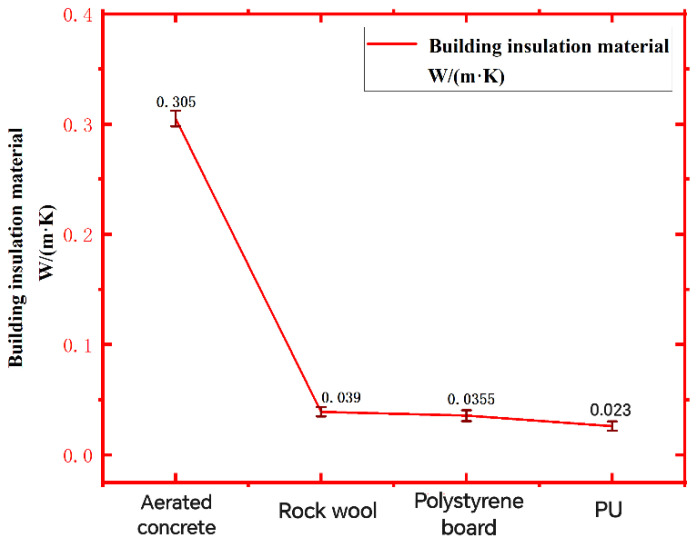
Comparison of thermal conductivity of different wall insulation materials.

**Figure 8:**
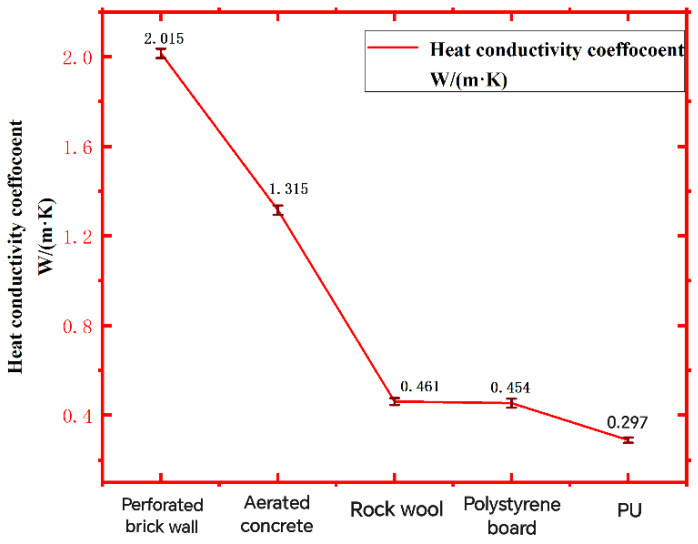
Comparison of thermal conductivity of different insulation walls.

Figure 7 is the comparison of the thermal conductivity of four kinds of insulation materials, and Figure 8 is the comparison of the thermal conductivity of the thermal insulation wall composed of porous brick walls and different insulation materials. Through the comparison of the data in Figure 6, it can be found that the thermal conductivity of recycled PU insulation materials is smaller than that of common insulation materials, with a thermal conductivity of 0.023 W/(m·K), and the thermal conductivity of the wall with insulation materials is smaller than that of traditional walls, including polystyrene (PS) board; The thermal conductivity of rock wool and recycled PU is small, and the thermal insulation performance is better. Figure 7 shows that the thermal conductivity of the recycled PU insulation wall is the smallest, and the thermal conductivity is 0.297 W/(m·K), which is the best thermal insulation effect.

Recycled PU has good thermal conductivity because it has a lower density, which allows it to contain more air or other gases per unit volume, and the thermal conductivity of these gases is much lower than that of solids, reducing the overall thermal conductivity of the material. The closed-cell structure can effectively limit heat transfer because the gas in the will not be exchanged with the outside world, forming a good thermal insulation layer [21,22,23,24]. PU is a polymer prepared by the reaction of isocyanate and polyol, and its molecular chain contains a large number of urethane links, which makes the polyurethane material itself have good elasticity and flexibility and also contributes to its thermal insulation performance [25]. Therefore, the low thermal conductivity of recycled PU is due to the combination of low density, closed-cell structure, chemical composition, and processing technology.

### 3.2. Analysis of Building Energy Consumption of Recycled PU Materials

The building is made of recycled PU material and is arranged using a fan coil unit system. The overall energy consumption of the building is simulated by Tianzheng HVAC software. The simulation results in Table 2 show that:

(1) The thermal properties of different wall insulation materials are different, which will have different effects on the total energy consumption of the wall [26]. The results also take into account the energy consumption of lighting and equipment, and the energy consumption of the wall with insulation material is reduced. Among them, the recycled PU material wall has the best energy-saving effect; with the traditional wall insulation effect, the recycled PU material wall can save 85.4% of energy consumption per square meter.
(2) The wall load of the wall with insulation materials is significantly reduced compared with the porous brick wall, aerated concrete insulated walls, rock wool insulation wall, polystyrene board insulation wall, and recycled PU material insulation walls, which can save 85.4% per square meter, in turn, 53.33%, 19.43%, and 16.26% energy consumption. It can be seen that the wall with insulation materials has improved the thermal insulation properties of the wall and reduced the energy consumption of the wall, among which the thermal insulation effect of the recycled PU material wall is the best, and the energy saving rate of the wall has reached 85.4%.

### 3.3. Economic Analysis of Recycled PU Materials

The economic analysis of recycled PU material building insulation walls mainly involves three aspects: material cost, construction cost and building economic energy consumption. Construction costs include labor costs, equipment leasing costs, and construction management costs [27,28]. Fees vary depending on the region and type of work. Therefore, this paper mainly analyzes the economy of building insulation walls from two aspects: material cost and building economic energy consumption.

#### 3.3.1. Cost of Recycled PU Materials

**Figure 9:**
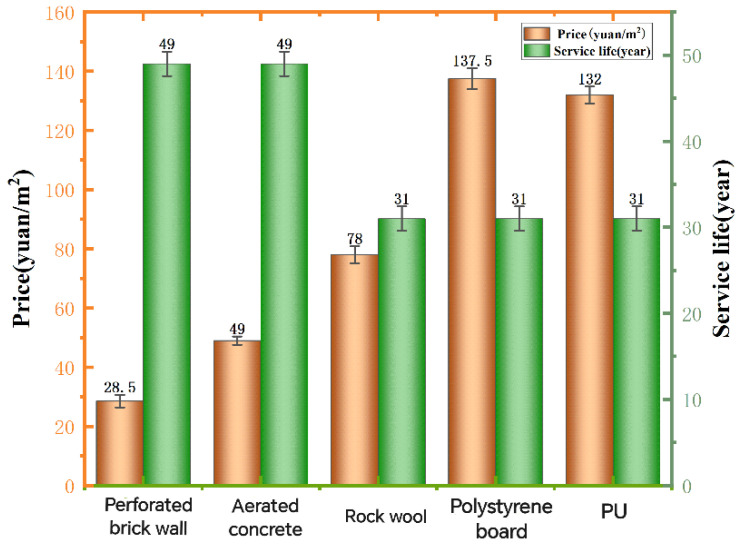
The price and service life of recycled PU material insulation walls.

The cost of recycled PU materials is the main part of the initial investment in the insulation wall, including the cost of the insulation material itself and the related accessories and installation costs. The average price of various wall insulation materials is calculated per square meter, the price calculation specification of perforated brick wall is 1000 mm × 240 mm × 1000 mm, the price calculation specification of insulation wall is 1000 mm × 70 mm × 1000 mm, and the service life of the wall is taken as the maximum service life, and the result is shown in Figure 9 below.

As can be seen from the table, the price and service life of PU are not as good as those of concrete compared with aerated concrete, but the thermal conductivity of the thermal insulation wall of the thermal insulation wall is 0.297 W/(m·K), and the thermal conductivity of the aerated concrete insulation wall is F = 1.33 W/(m·K). Although the initial investment is higher than that of concrete, the gap between the initial investment will be slowly balanced in the cost of refrigeration equipment and electricity, and the recycled PU material insulation wall has a better living experience. Compared with aerated concrete, rock wool insulates walls and polystyrene board insulation walls. The thermal conductivity of the three recycled PU material insulation walls is not much different, and all have good building insulation effects. However, the service life of stone wool is shorter, and the price of polystyrene board is more expensive. Based on the above analysis, it is concluded that the recycled PU material insulation wall has a long service life and low price while having a good thermal insulation effect, and the total load is the most cost-effective.

#### 3.3.2. Analysis of Energy-Saving Benefits of Recycled PU Material Insulation Wall

The energy-saving benefits of recycled PU insulation walls are considered from a long-term perspective. Thermal insulation walls can effectively reduce the energy consumption of buildings, reducing the cost of heating in winter and cooling in summer [29,30,31]. At the same time, the insulated wall can also reduce carbon emissions, and while the building is insulated, it also has green environmental protection characteristics [32].

(1) Total Life Cycle Investment Costs

The total investment cost for the whole life cycle of a building insulation wall includes the initial investment cost of materials and the energy cost of maintaining the annual heating and cooling of the room within the thermal comfort range [33,34]. The calculation formula is as follows:

(1) $$C_{C}=C_{I}+C_{E}PWF$$

(2) $$C_{E}=\frac{Q}{3600COP}\;c_{e}$$

(3) $$WF=\frac{1}{i-g}\left[1-{\left(\frac{1+g}{1+i}\right)}^{N}\right]$$

*C_C_*—The total input cost in the whole life cycle, yuan/m^2^;

*C_I_*—The initial investment cost of the insulation wall, yuan/m^2^;

*C_E_*—The energy cost of maintaining the annual heating and cooling of the room within the thermal comfort range, yuan/m^2^;

*Q*—Total annual heating and cooling loads, KJ/m^2^;

*c_e_*—Electricity cost (no peak and valley electricity), 0.6 yuan/KWh;

*COP*—Coefficient of performance of air conditioning system, take 2.5;

*g*—Inflation rate, take 7.5%;

*i*—Bank rate, take 2%;

*PWF*—Present value coefficient;

*N*—The service life of each insulation material (take the maximum service life).

**Figure 10:**
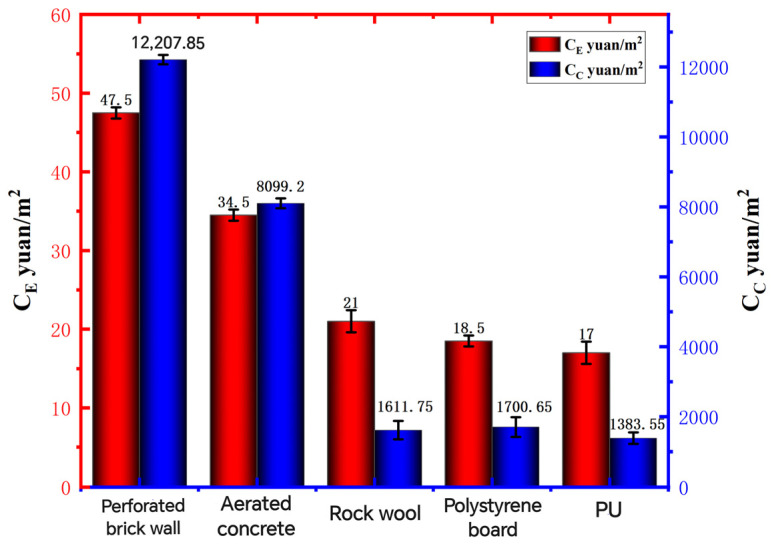
Energy costs and life-cycle input costs.

Through the calculation of the above formula, the result is shown in Figure 10 below:

As can be seen from Figure 10, the annual heating and cooling costs of different insulation walls in maintaining the indoor thermal comfort range are different, and the prices show a cliff-like decline. The energy consumption cost of the porous brick wall without the addition of insulation materials is the highest, and the energy consumption cost of the aerated concrete insulation wall is lower than that of the porous brick wall, but the cost is still very high. Compared with the first two, rock wool insulates the wall. The energy consumption cost of polystyrene board insulation wall and recycled PU insulation wall is relatively low, and the energy consumption cost of recycled PU insulation wall is the lowest, with an annual energy consumption cost of 16 yuan/m^2^. Compared to perforated brick walls, aerated concrete insulated walls and rock wool insulation walls, the annual energy consumption cost of polystyrene board insulation walls and recycled PU insulation walls was reduced by 66.12%, in turn, 54.29%, 20.0%, and 15.79% year over year. The annual energy consumption cost of recycled PU insulation wall is low because the thermal conductivity of recycled PU insulation material is very low, which has a better thermal insulation effect compared with ordinary wall insulation materials, which greatly reduces the energy consumption of the building, so the energy consumption cost is more economical. From the perspective of the total investment cost C_C_ in the whole life cycle of different insulation walls, the total investment cost of recycled PU insulation walls is still the lowest. Compared with other building insulation walls, the total investment cost of the whole life cycle of recycled PU insulation walls was reduced by 89.54% at one time, in turn, 84.55%, 10.99%, and 15.5% year over year. Although the price of recycled PU insulation wall is not as good as that of some other insulation materials, due to its low energy consumption performance, the total investment cost is still lower than that of other building insulation walls even if it has a high price, which also explains the reason why the price of recycled PU material is high, but it is still selected as the optimal wall insulation material. Generally speaking, the initial investment of recycled PU non-thermal insulation walls may be higher than that of some thermal insulation walls because of their excellent thermal insulation performance. This saves significant costs in terms of building energy consumption, so the total investment cost in the whole life cycle and the energy cost of maintaining the annual heating and cooling of the room in the thermal comfort range are more economical than those of other thermal insulation walls.

(2) Analysis of environmental benefits

Environmental protection refers to the protection effect of products or materials on the environment during use, including resource recycling; waste disposal, energy conservation and emission reduction [35,36,37]. At present, the general treatment methods for waste insulation materials are incineration and burial [38]. This will cause different degrees of pollution to the environment, and the solid waste recycling process of recycled PU insulation materials is now very perfect, and we can realize solid waste recycling through chemical degradation and refoaming. In addition to solid waste recycling, carbon emissions from materials are also a major criterion in the evaluation of environmental benefits. We use the following formula to calculate the carbon emissions of different insulation walls:

(4) $${AM}_{AV}=ES*EF$$

(5) $$ES=\frac{Q}{3600COP}$$

(6) $$F_{T}={AM}_{AV}*PWF$$

*AM_AV_*—Annual CO_2_ emissions, kg/(year·m^2^);

*ES*—The energy consumed by the total annual heating and cooling load, kWh/(year·m^2^);

*EF*—Carbon emission factor, kgCO_2_/kg standard coal, take 3.18 kgCO_2_/kg standard coal;

*F_T_*—Carbon emission tax, yuan/kgCO_2_, take 0.12 yuan/kgCO_2_.

**Figure 11:**
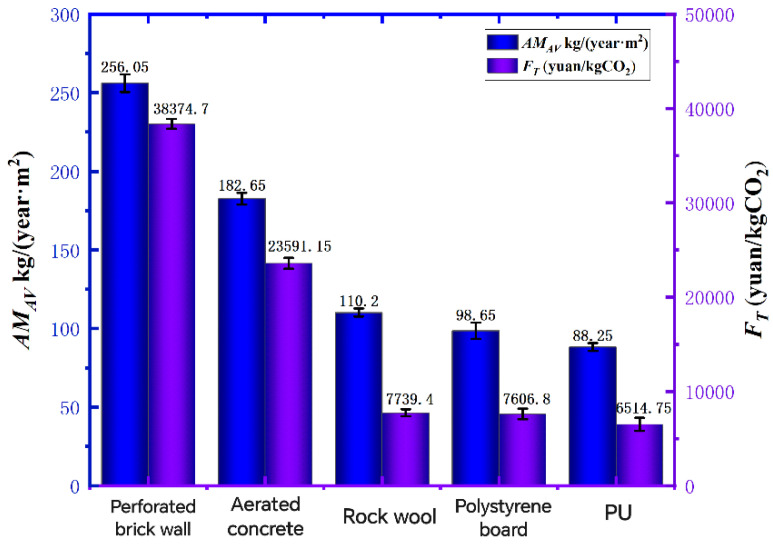
Annual CO_2_ emissions and carbon emissions tax graph.

From the data in Figure 11, the annual CO_2_ emission of porous brick wall is the highest, which is 252.1 kg/(year·m^2^), and the annual CO_2_ emission of recycled PU insulation wall is the lowest, which is 86.5 kg/(year·m^2^), because recycled PU insulation wall has better thermal insulation performance than porous brick wall, so that its annual heating and cooling total load consumes less energy, so the carbon emission is the lowest, and compared with porous brick wall; aerated concrete. The annual carbon dioxide emissions of rock wool and polystyrene boards and recycled PU material insulation walls decreased by 65.69% year-over-year, in turn, 53.32%, 20.2%, and 15.45%. It can be seen that the recycled PU material insulation wall has the advantages of high thermal insulation performance and low carbon emissions, which is in line with the national policy of “carbon peak and carbon neutrality”. Under this excellent carbon reduction ability, the carbon emission tax of recycled PU insulation walls is also the lowest compared with that of porous brick walls and aerated concrete. The rock wool and polystyrene boards and recycled PU insulation walls saved 84.44%, in turn, 73.99%, 20.09%, and 16.42% of carbon tax.

Through the comparison of annual carbon dioxide emissions and carbon emission taxes, recycled PU insulation walls have better environmental protection effects. Coupled with the energy-saving effect of recycled PU materials demonstrated above, we can conclude that recycled PU insulation walls have more efficient energy-saving and emission-reduction capabilities than ordinary insulation walls.

## 4. Concluding Remarks

In this paper, a new type of building insulation material, recycled PU, was used as the wall insulation material, and a new type of building insulation wall was successfully prepared. In terms of the price of its raw materials, service life, building energy consumption, and whole life-cycle input costs, a multifaceted comparison of annual CO_2_ emissions and carbon taxes leads to the following conclusions:

The thermal conductivity of recycled PU is smaller than that of common building insulation materials on the market, with a thermal conductivity of 0.023 (W/(m·K)), and the building insulation wall made of it has higher thermal insulation performance and the thermal conductivity of recycled PU insulation wall is 0.297 (W/(m·K)). Although the price per square meter of recycled PU insulation material is higher than that of some other materials, it has the best comprehensive cost performance due to its high thermal insulation energy-saving performance and long service life.

Compared with the traditional insulation wall, the new building insulation wall using recycled PU as the wall insulation material has higher thermal insulation performance, which can save 85.4%/m^2^ of energy consumption compared with the traditional double-sided plastered brick wall, reduce the annual energy consumption cost by 66.12%/m^2^, reduce the total investment cost of the whole life cycle by 89.54%/m^2^, reduce the annual carbon dioxide emissions by 65.69% m^2^ year over year, and saves 84.44%/m^2^ from carbon emission tax. It has high energy-saving benefits and excellent environmental performance.

Therefore, the new type of building insulation wall has excellent energy conservation and environmental protection and provides a new way to achieve the goal of building energy conservation and emission reduction.

## Figures and Tables

**Table 1:** Indoor and outdoor parameters.

Summertime			Calculate the dry-bulb temperature outside the air conditioner			34.2 °C	
Average outdoor wind speed						1.5 m/s	
Calculate the (dry-bulb) temperature outside the ventilation room						31.8 °C	
Room type	Temperature		Humidity		Lighting power	Device power	Personnel density
Living quarters	summertime	winter	summertime	winter	15 W/m^2^	20 W/m^2^	4 person
	24 °C	22	60–65%	40–50%			

**Table 2:** Simulation of building energy consumption for different insulation walls.

Heat Preservation Materials	Total Energy Consumption (kW·h/m^2^)	Cooling Energy Consumption (kW·h/m^2^)	Energy Consumption for Heating (kW·h/m^2^)	Equipment Energy Consumption (kW·h/m^2^)	Lighting Energy Consumption (kW·h/m^2^)
Ordinary brick walls	198.2	87.7	64.8	13.9	31.8
Aerated concrete	145.7	57.5	42.5	13.9	31.8
Rock wool	84.4	21.1	15.6	13.9	31.8
Polystyrene board	81.2	20.4	15.1	13.9	31.8
Recycled PU board	68	12.8	9.5	13.9	31.8

## Data Availability

The data presented in this study can be provided at the request of the corresponding author due to industrial privacy concerns.
